# Mechanisms That Govern Recombinase Fidelity Control During Eukaryotic Homologous Recombination

**DOI:** 10.1002/bies.70173

**Published:** 2026-08-13

**Authors:** Ilayda Korkmaz, J. Brooks Crickard

**Affiliations:** ^1^ Department of Molecular Biology and Genetics Cornell University Ithaca New York USA

**Keywords:** Dmc1, homologous recombination, Rad51, strand exchange

## Abstract

Homologous recombination (HR) is a DNA double‐strand break repair pathway that preserves genome integrity by restoring genetic information lost through programmed or spontaneous DNA damage. As a template‐directed process, HR identifies homologous DNA sequences to accurately repair broken chromosomes while minimizing inappropriate recombination events. Central to this process are the RecA‐family recombinases. Most eukaryotes use Rad51 during mitosis and meiosis and Dmc1 during meiosis to locate and pair homologous DNA sequences. To ensure high‐fidelity repair, eukaryotes have evolved regulatory protein networks that control recombinase filament assembly, organization, and strand exchange. Here, we review recent advances in understanding how recombinase filament length, architecture, and dynamics influence the fidelity and outcome of homologous recombination. We discuss their distinct roles in mitotic and meiotic recombination and propose how evolution has shaped filament properties to regulate interactions between donor and recipient DNA templates and promote accurate genome maintenance.

## Graphical abstract

The rate of recombinase filament formation controls the fidelity of the homologous recombination reaction by either restricting the length of strand pairing for high‐fidelity repair or increasing the length of strand pairing for more complex outcomes.

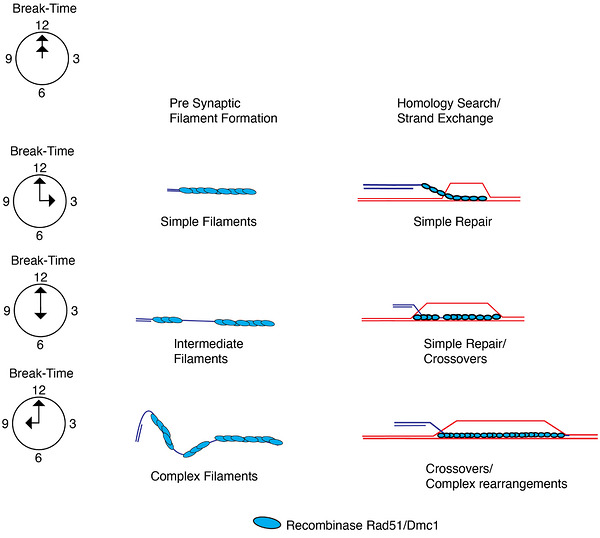

## 1 Introduction

**FIGURE 1:**
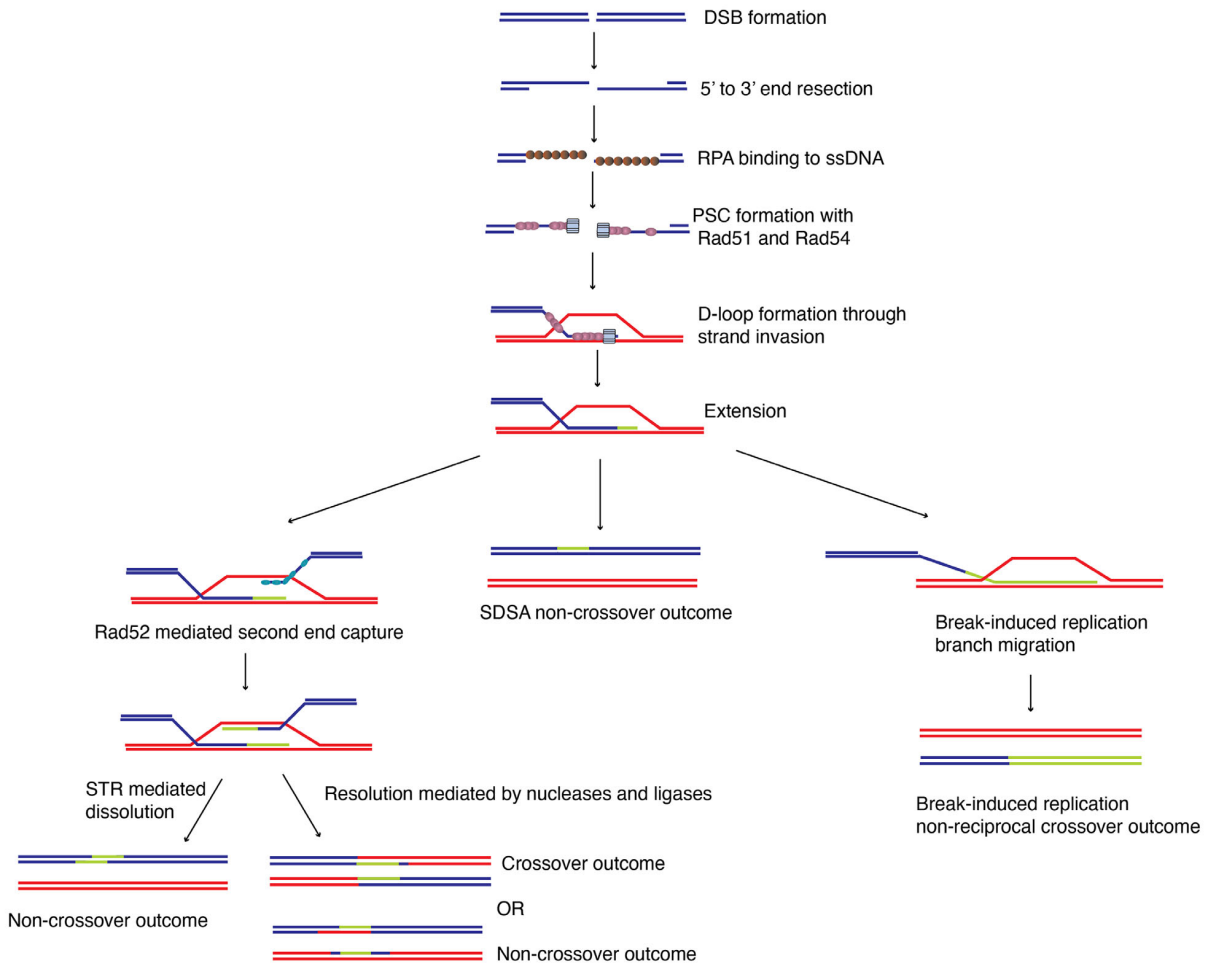
Basic homologous recombination pathway. A general schematic illustrating the general recombination pathway. PSC stands for presynaptic complex composed of Rad51, Rad54, and ssDNA.

Homologous recombination is a template‐based repair pathway that is predicated on finding a matching piece of DNA to repair a double‐strand break (DSB) [1]. This pathway begins by resecting the break into a single‐stranded DNA fragment that can then act as a recipient DNA in the reaction (Figure 1). The Mre11‐Rad50‐Xrs2 complex initiates resection in *Saccharomyces cerevisiae*. This activity is done by the MRE11‐RAD50‐NBS1 complex in humans [2, 3]. These complexes act to tether the DNA, remove problematic DNA ends, and promote short‐range resection [2, 3]. Long‐range end resection occurs as the result of two semi‐redundant enzymatic pathways and is facilitated by either exonuclease 1 (ExoI) or the Sgs1‐Top3‐Rmi1‐Dna2 complex (Bloom‐TOP3‐RMI1‐RMI2‐DNA2 in humans) [2, 4]. During meiosis, only Exo1 participates in end resection [5, 6]. The resected ssDNA is first covered by Replication protein A (RPA), a single‐strand DNA‐binding protein that protects the DNA from further breaks and activates the DNA checkpoint kinases [7]. RPA is then replaced by recombinases of the RecA family. These recombinases form filaments on the ssDNA and, with the assistance of motor proteins, form the presynaptic complex (PSC). These filaments use the ssDNA as a guide to search the genome for a matching sequence element to be found in either the sister chromatid or the homologous chromosome. This search is biased toward the sister chromatid in mitotic repair and toward the homologous chromosome in meiotic repair. Still, both templates can be used during both lifecycle phases in diploid organisms.

The search for a matching DNA sequence is a process called the homology search and is facilitated by members of the RecA family of proteins [8, 9]. These enzymes not only facilitate the exchange of DNA strands but also aid in the recruitment and organization of factors that assist in the identification and selection of the homologous sequence [9, 10, 11, 12, 13, 14]. Once a suitable homology has been found, a displacement loop (D‐loop) forms, and strand exchange occurs. The D‐loop structure primes DNA synthesis and restores any information that was lost at the initial break [15, 16] (Figure 1).

The extension of the recipient DNA creates sequence homology to the other end of the DSB, known as the second end. The completion of repair can occur through several discrete pathways that depend on utilizing the second end of DNA. If disruption of the D‐loop occurs before the annealing of the second end, then the reaction will proceed through synthesis‐dependent strand annealing (SDSA) or break‐induced replication (BIR) [17, 18]. Alternatively, the second end of DNA can be captured before D‐loop disruption. This results in the formation of Holliday junctions [19, 20, 21]. If no end is found, repair will be handled through BIR (Figure 1).

Holliday junction intermediates can be cleared through resolution or dissolution. Both outcomes initiate through branch migration of the newly formed DNA joints [22, 23, 24, 25, 26]. Dissolution occurs when the Sgs1‐Top3‐Rmi1 (STR) complex drives branches together [22, 23], followed by decatenation of the DNA through the topoisomerase activity of Top3‐Rmi1 [27, 28, 29]. Dissolution prevents the exchange of information between the donor and recipient DNA. In contrast, Holliday junction resolution occurs through cleavage and ligation by structure‐specific nucleases and a ligase. Resolution can result in the exchange of DNA strands between the donor and the recipient DNA. This outcome can be referred to as a crossover. However, if DNA is not exchanged, the outcome is a non‐crossover.

This is a general overview of the homologous recombination pathway with eukaryotic proteins. The remainder of this review will focus on specific aspects of how the length of recombinase filaments, both pre‐ and post‐synapsis, regulates the fidelity of the recombination reaction. We will discuss both in the context of mitotic and meiotic DNA repair.

### 1.1 RecA Family Filament Length and Fidelity

In all domains of life, the recipient ssDNA is bound by a member of the RecA family of recombinases, known as RecA in eubacteria, RadA in archaea, and Rad51/Dmc1 in eukaryotes. On the protein sequence level, RecA from eubacteria is about 20% identical to Rad51/Dmc1 and around 20% identical to RadA from archaea [30, 31, 32, 33]. In contrast, the sequence identity between RadA and Rad51/Dmc1 is 40%–50%, implying a closer evolutionary relationship between archaeal and eukaryotic recombinases than between their eubacterial and eukaryotic counterparts. Rad51 and Dmc1 arose from a gene duplication event of an ancestral recombinase early in the eukaryotic lineage [32]. They have since undergone specialization.

Recombinase enzymes in different domains share structural similarities that indicate conservation of the recombination reaction [34, 35, 36]. Structurally, all members of the RecA family of recombinases form helical filaments on the recipient DNA, stretching it by 1.5 times the contour length of B‐form DNA [37]. ATP is required for filament formation, and the ATP is shared between adjacent RecA protomers. The filament forms a helical pitch that can vary from 90 to 120 Å, depending on the state of the nucleotide cofactor. Conversion of ATP to ADP results in the formation of filaments with longer helical pitch, which are less stable on DNA [38, 39]. The stretched structure is essential for creating tension on the DNA, which, when relaxed, makes the strand pairing reaction thermodynamically favorable.

The growth of recombinase filaments on ssDNA is a tightly regulated process that has a significant impact on the fidelity of the reaction. A general misconception is that the length of the resected ssDNA correlates one‐to‐one with individual filament length or the number of protomers in a contiguous filament. This is likely not the case, and while recombinases can exist as longer filaments, they can also form short interspersed filaments on the ssDNA [40]. The flexibility provided by ssDNA linker regions between interspersed filaments improves the speed of the homology search by allowing intersegmental transfer in 3D and increased contact potential along the donor [11]. During repair, the resection of the recipient DNA allows for the formation of more recombinase filaments. Extensive recombinase filament formation can also be problematic, creating the potential for hyperrecombination events in the form of DNA multi‐invasions [41, 42, 43]. These hyperrecombination outcomes can cause catastrophic rearrangements within the genome. Therefore, restriction of the RecA/Rad51 filament size or density on ssDNA is important in maintaining fidelity during homologous recombination.

In *S. cerevisiae*, the nucleation and extension of Rad51 filaments occur through a coordinated effort of Rad52 and Rad55/57 [44], with Rad52 acting to nucleate filaments and Rad55/57 acting to extend them [44, 45, 46]. Additionally, there are contributions from other Rad51 paralogs, Psy3 and Csm2, as part of the Shu complex [47, 48]. In higher eukaryotes, the RAD51 mediator BRCA2 works in coordination with the RAD51 paralogs, including RAD51B, RAD51C, RAD51D, XRCC2, and XRCC3 [49, 50, 51, 52, 53] to regulate the formation of RAD51 filaments. In both systems, these proteins work to coordinate the length of Rad51 filaments. The specific mechanisms underlying this regulation are an ongoing area of research.

While these complexes work to build Rad51 filaments, there are helicase/translocase proteins that actively work to disassemble Rad51 filaments. The best‐characterized system for visualizing the reversal of Rad51 filament formation is in *S. cerevisiae* and involves the helicase Srs2 [54, 55, 56, 57, 58, 59, 60]. This SF1‐type helicase can promote the disruption of Rad51 filaments on ssDNA. Based on mutagenesis studies, the regulation of Rad51 filament lengths is the primary role of the helicase during the recombination reaction [54]. Rad51 filaments may undergo multiple rounds of disassembly and reassembly to facilitate long‐range pairing across the genome. By preventing the runaway growth of Rad51 filaments, Srs2 prevents the accumulation of toxic intermediates and improves the overall fidelity of the recombination reaction [61].

During meiosis, Dmc1 disassembly is unaffected by the activity of Srs2 [62, 63]. However, assembly may be affected by Srs2 because Dmc1 filament formation is dependent on Rad51. Together, Dmc1 and Rad51 form isolated interspersed filaments both in vitro and in vivo [64, 65, 66]. These cooperative filaments form on both sides of the double‐strand break, with Dmc1 occupying the 3’ end of the ssDNA [65, 67]. It is currently unclear if the length of Dmc1 filaments is modulated by disassembly or if they are simply controlled through assembly by factors such as the Mei5‐Sae3 complex in budding yeast [68, 69, 70] and BRCA2 in the human system [71].

### 1.2 Off‐Pathway Binding Is a Problem

The formation of recombinase filaments on undamaged dsDNA through nonspecific binding is also a problem, which can become toxic by impairing chromosome segregation [72]. Eubacteria and eukarya have developed different strategies to deal with this problem. In bacteria, RecA has poor affinity for dsDNA, and competition for binding between ssDNA and dsDNA is not an issue [73]. High rates of ATP hydrolysis further ensure that dsDNA binding does not cause significant problems because it limits the lifetime of binding [74]. In contrast, eukaryotic recombinases have comparable affinity for both dsDNA and ssDNA and slow ATP hydrolysis rates, which can lead to filaments being stuck on dsDNA. These interactions are generally considered off‐pathway interactions [74], but have also been thought to mimic the post‐synaptic structure at the D‐loop. Furthermore, inappropriate binding of Rad51 or Dmc1 to dsDNA can also reduce the available pools of recombinase proteins required for effective recombination. Therefore, modulation of recombinase binding to dsDNA is important in ensuring an effective recombination reaction. DNA motor proteins of the Snf2 family remove Rad51 and Dmc1 from dsDNA. The proteins included in this group are Rad54 (RAD54L), Rdh54 (RAD54B), and Uls1 (RAD54L2) [72].

Rad54 and Rdh54 are paralogs that can use their motor function to directly remove Rad51 from dsDNA [75]. Rdh54 does this under non‐repair conditions but likely contributes to the dynamic equilibrium of Rad51 during HR. Rdh54 is the only translocase that is known to remove Dmc1 from dsDNA [76, 77]. Rad54 is significantly more active during strand pairing and can act to remove Rad51 from the 3’ end of the D‐loop to promote DNA synthesis [78]. The role of Rad54 will be discussed more below. Uls1 is a Snf2 ATPase with a SUMO‐dependent ubiquitin ligase domain that controls Rad51 levels by promoting degradation of SUMOylated Rad51 and is thought to act in the later stages of HR based repair [79]. Together, these Snf2 motors remodel filaments of Rad51, preventing pathogenic binding to dsDNA and reducing chromosome loss. Future work should focus on crosstalk between these remodeling enzymes during the HR reaction.

### 1.3 Processivity of Strand Pairing

Homology search depends on the dynamic on/off rate of recombinase filaments on donor DNA. The length of pairing promoted by individual recombinase filaments is the earliest factor that controls the overall length of strand pairing between recipient and donor DNA. Each RecA/Rad51/Dmc1 protomer can bind three nucleotides of DNA, which are separated by the DNA‐binding domains. Stretching of the DNA allows triplet base flipping of the donor DNA, which can then be recognized by the incoming strand [36, 37, 49, 80, 81]. Pairing of the incoming strand and relaxation of the stretched donor DNA by hybridization results in a thermodynamically favorable event. The nonhomologous or outgoing strand of DNA is stabilized by a secondary DNA‐binding site that temporarily increases the favorability of strand separation by preventing re‐annealing of the parent donor duplex [64, 82, 83, 84]. Eight nucleotides are required for kinetic sampling of the bases, and twelve nucleotides are required for stable selection of a homologous sequence [85, 86, 87]. In RecA filaments, pairing can now proceed over thousands of nucleotides [1], and strand pairing is highly processive. This is not the case with eukaryotic recombinases, and Rad51 and Dmc1 are less processive during strand pairing [88, 89]. It is unclear if processive pairing occurs to the end of an individual filament or if it can be interrupted, or the role of ATP hydrolysis in the pairing process.

**FIGURE 2:**
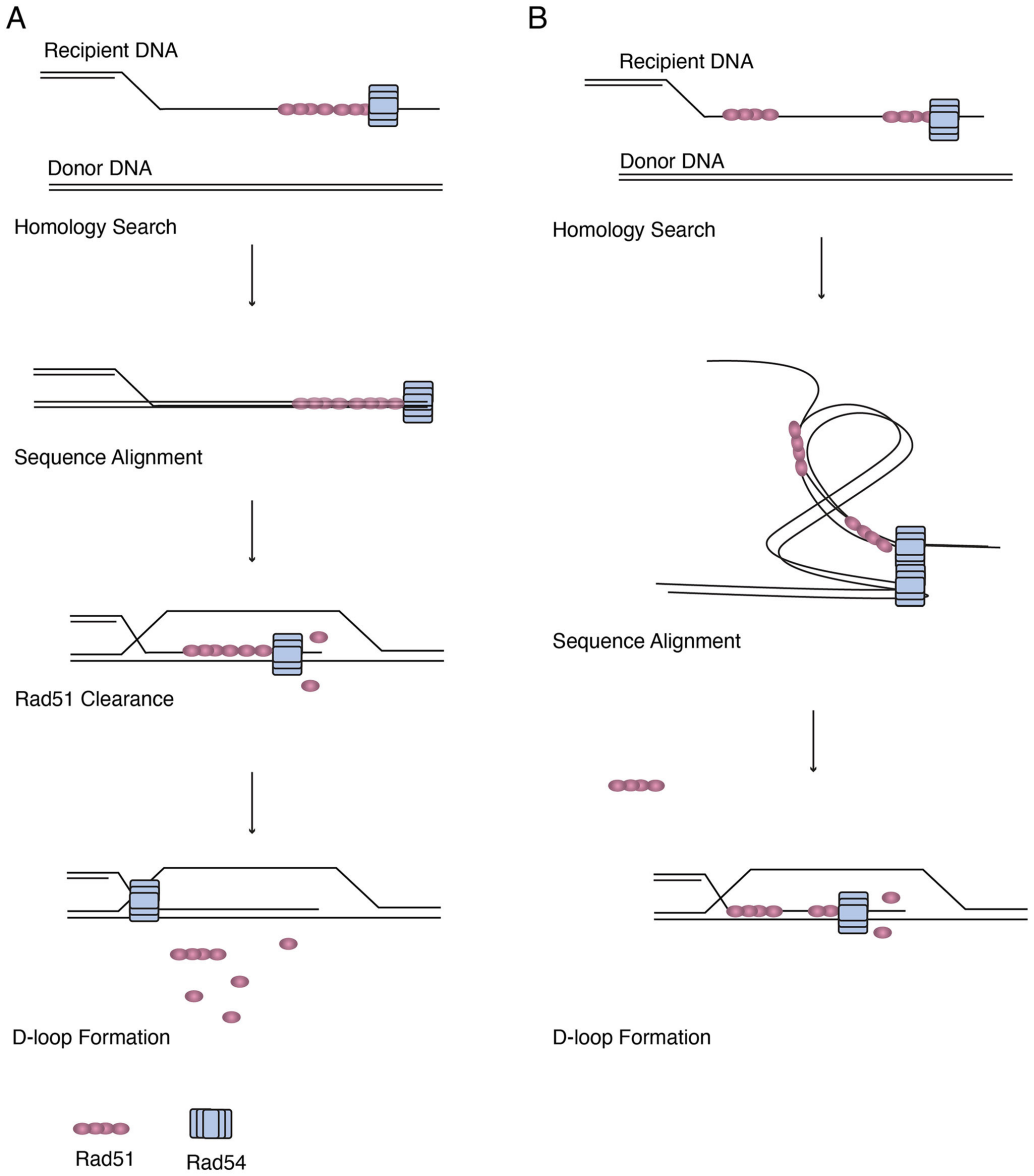
Models for Rad54‐mediated D‐loop formation. (A). Model for Rad54 mediating processive pairing activity of Rad51 to promote D‐loop formation. (B). Model for Rad54‐mediated D‐loop formation through loop extrusion.

The difference in processive strand pairing between RecA and Rad51 has precipitated the need to explain how Rad51 can promote stable strand pairing products. It is believed that RecA can facilitate the formation of longer DNA pairing tracts because it is able to hydrolyze ATP at a rate that prevents autoinhibition of the recombination reaction [87]. In contrast, the ATPase activity of Rad51 is too slow to prevent this. A proposed solution to this in eukaryotes is the contribution of Rad54 to the pairing reaction. Rad54 is known to disrupt filaments of Rad51 and to promote DNA branch migration [33, 90]. The hypothesis is that physical translocation of the motor will then promote pairing by inducing ATP hydrolysis of Rad51, and the pairing tracts will be proportional to the processivity of the Rad54 motor along the donor DNA [91] (Figure 2A). By disrupting monomers of Rad51, Rad54 can provide the same role as the high hydrolysis rate of RecA, making the pairing reaction conserved between eubacteria and eukarya. This model provides a simple solution to stabilizing D‐loops in eukaryotes and creating accessibility of the 3’ end to DNA polymerases [33].

However, an alternative model exists. Recently, it has been shown that Rad54 can extrude loops of DNA, which are slightly underwound [92, 93, 94]. Rad51 has a higher affinity for underwound DNA, and the extrusion of a donor DNA loop could provide a platform for multiple short filaments of Rad51 to pair with the donor [92]. Short and interspersed filaments could have the same thermodynamic stability as a significantly longer Rad51 filament while gaining higher three‐dimensional flexibility. The combination of short filaments will apply additive torque to the donor DNA as the DNA strands are exchanged [92]. This accumulated torque can lead to DNA melting and D‐loop formation (Figure 2B). By creating a looped structure to allow short filament cooperation, the filaments may be easier to disassemble, preventing extensive contact between the donor and the recipient. In this model, Rad54 can still dislodge Rad51 from dsDNA to create accessibility of the 3’end, but it does not have to translocate along the donor to create extended pairing tracts. Instead, pumping of a loop provides a platform for stable pairing products [68, 95]. Ultimately, the separation of these two models will require mutants that isolate the different functions of Rad54.

In meiosis, Rad51 and Dmc1 both filament on the resection product to search for homology, with Dmc1 responsible for the main recombinase activity [64, 65, 66]. Interestingly, Rad54 is blocked from binding Rad51 in the early stages of budding yeast meiosis [96, 97] and is not required for Dmc1‐mediated strand exchange. In humans, RAD54L expression is reduced in germline cells, suggesting a similar Rad54 activity reduction. The reduction in Rad54‐mediated catalysis of strand pairing during the onset of meiotic recombination may reflect the timing required for recombinase filaments to mature into the longer structures. This delay may create a kinetic window that is better for homologous chromosome pairing, as longer structures lead to stronger bridging between the chromosomes. During meiosis, Dmc1 is also aided by the Hop2‐Mnd1 [98] complex for strand pairing, which helps maintain contact with the donor. Rad54 acts later in meiosis. This activity is likely due to cleaning up double‐strand breaks that need to be repaired but do not form crossovers [99]. Future work will be needed to understand the role of Rad54 in promoting clean‐up repair during meiosis.

### 1.4 The Controversy of Rad51 and Dmc1

All RecA homologs contain two DNA‐binding sites that act collectively along the filament. DNA‐binding site I comprises two DNA‐binding loops, L1 and L2, that stabilize the triplet stack in the recipient DNA and intercalate the donor duplex to facilitate base‐pairing by the incoming strand [100]. Previously, a lack of defined electron density of the L2 in most high‐resolution structures of Rad51/Dmc1 had prompted the interpretation that this region is mobile during subsequent steps in the strand exchange reaction. A recently published structure has been able to solve the L2 structure, which shows high conformational heterogeneity during strand pairing [101]. Of interest is that this region represents one of the few differences between Rad51 and Dmc1. Previous studies have swapped either L1, L2, or both between Rad51 and Dmc1 [102, 103, 104, 105]. Additionally, specific amino acid substitutions have also been switched between the two recombinases. Interestingly, the Dmc1 binding loops are more efficient at stabilizing mismatches during strand exchange [106]. Swapping these amino acids results in a shift in activity, with the strand exchange activity of Rad51 becoming more like Dmc1 and vice versa [102, 103, 105].

The biochemical difference in mismatch tolerance that was demonstrated in the amino acid swapping experiments has come under heavy scrutiny because it is unclear what the selective pressure is that would drive a difference in mismatch tolerance between the two recombinases. More recent work has revealed that Dmc1 also has faster strand exchange kinetics than Rad51 [102], suggesting that the pairing of nucleotides may be more continuous. A potential update to the existing model is that the interaction between L1 and L2 acts as a gate to restrict nucleotide pairing. This could partially explain the apparent mismatch tolerance difference between Rad51 and Dmc1, as a faster, more processive reaction will gain enough new base pairs to stabilize the pairing event even in the presence of nucleotide mismatches. This kinetic difference may allow for more stable connections to form between two pieces of DNA. In the case of Dmc1, this may lead to decreased recombination fidelity. The impact of this difference has not been fully explored in vivo.

The evolutionary reason for the difference in pairing between Rad51 and Dmc1 is still unclear. However, Archaeal RadA more closely resembles Dmc1, and there is evidence that suggests Rad51 may have evolved higher stringency in eukaryotes [102, 103, 107]. This hypothesis suggests that lower fidelity in Dmc1 is not selected against despite appearing to be a shortcoming, because the high fidelity of HR is one of its key advantages over other DSB repair mechanisms. Instead, it is conserved and contained to its useful specialized role during meiosis to generate genetic diversity. Alternatively, Dmc1 may form longer pairing tracts on DNA, and this may be favorable for forming connections between chromosomes. This creates a scenario where strand pairing length may be selected against during mitosis and selected for during meiosis. Future work should focus on fully explaining this model.

### 1.5 Righting a Wrong

Intrinsic to increasing the rate of D‐loop formation is an increased potential to promote mismatches between the donor and recipient DNA during strand pairing. The incorporation of mismatches can challenge the fidelity of the DNA repair reaction. We have discussed how fidelity is maintained before and during the early stages of pairing. However, sometimes mis‐paired DNA makes it past the earlier quality control checks, resulting in the formation of homoeologous heteroduplexes. Below, we will discuss how homoeologous recombination is reversed and the interaction between recombinases and proteins that facilitate the reversal of strand pairing.

First, we will discuss how the mismatch repair (MMR) family of proteins interfaces with HR to promote high‐fidelity repairs. The structures and functions of this family are highly conserved across prokaryotes, eukaryotes, and even viruses [108, 109, 110, 111]. In bacteria, the members of the mismatch repair proteins that have been implicated in HR consist of MutS and MutL. In *S. cerevisiae*, the relevant homologs of these proteins can be further divided into MutS alpha (Msh2‐Msh6), MutS beta (Msh2‐Msh3), MutL alpha (Mlh1‐Pms1), and MutL gamma (Mlh1‐Mlh3) [112]. These complexes function as obligate heterodimers and carry out discrete functions during mismatch repair. Typically, these complexes are designed to identify errors that form during DNA replication [113, 114, 115].

The earliest evidence for their role in HR stems from interference in RecA‐mediated strand transfer of homoeologous DNA, or donor DNA that is not fully homologous to the recipient [116]. This work showed that base pairing in RecA‐mediated heteroduplexes is more tolerant of mismatches than B‐form DNA base pairing in vitro. However, RecA‐mediated recombination prefers fully homologous substrates in vivo, indicating that there are other factors that reverse homoeologous recombination [117]. One explanation is the MMR family of proteins. In vitro strand exchange assays using *E. coli* proteins have shown that MutS can inhibit strand exchange and block branch migration [116]. This activity is augmented by MutL [116]. In this case, MutL may aid MutS by stabilizing its tetrameric form, a more effective regulatory structure [118, 119, 120]. MutL also increases the dwell time of MutS on a mismatch from seconds to minutes [121], and this may impact its ability to regulate downstream recombination intermediates.

MutL can also limit de novo extension of strand exchange intermediates by blocking the 3’ end of an invading strand in a D‐loop, preventing DNA polymerase access [122]. MutS‐MutL together have been shown to trap the recombination intermediate and recruit UvrD to unwind it with MutL ATPase activity, making the unwinding unidirectional [119]. In yeast, Srs2 is an ortholog of UvrD [123]. MutS and MutL likely do this by inducing rotational constraints on DNA and preventing heteroduplex extension [124]. A mismatch‐bound MutS‐MutL D‐loop is more stable, likely waiting for another protein, such as a helicase, to come in and reverse the strand exchange [124]. Msh2‐Msh6 recognizes base‐base mismatches and small insertion‐deletion loops, while Msh2‐Msh3 has a higher affinity for larger insertion‐deletion loops [114, 125]. Msh3 and Msh6 are likely to be the subunits in their respective complexes that recognize the mismatch due to their higher levels of conservation of the DNA‐interacting motifs of MutS compared to Msh2 for mismatch repair [126]. How this impacts mismatches during strand pairing and their interaction with recombinases remains unclear. Human MSH2‐MSH6 has been shown to interact with model D‐loop structures [127]. The contribution of Rad51 in this process remains unknown. However, the efficiency of Rad51 removal from DNA remains a leading hypothesis, with more efficient removal resulting in higher fidelity.

### 1.6 Disruption of Nascent D‐Loops

Most events during HR occur through a three‐strand intermediate known as the D‐loop (Figure 1). Recent work has focused on understanding the reversibility of these structures in *S. cerevisiae*, focusing on how helicases contribute to the general rejection of fully paired duplexes [41, 128]. These helicases work either during the initial formation of the D‐loop or after the D‐loop has been extended by DNA replication. The rate of reversal will be impacted by the length of pairing between the donor and the recipient and the presence or absence of recombinases.

In *S. cerevisiae*, the helicases Sgs1, Mph1, and Srs2 contribute to dynamically regulating the D‐loop. The ortholog of Mph1 is FANCM in higher eukaryotes, while the activity of Sgs1 is separated into multiple RecQ helicases, including Bloom (BLM), RecQ5, Werner (WRN), and RecQ4 [129]. There is no known direct homolog of Srs2 in the human system. However, FBH1 is functionally similar [118, 130]. These helicases operate through kinetic competition, dynamically regulating the efficiency of D‐loop capture. Several studies suggest that Mph1 may have a greater impact on the disruption of extended D‐loops, while Sgs1 may regulate the nascent D‐loop through its interaction with Top3‐Rmi1 [128, 131]. These findings were built on earlier biochemical and genetic studies examining the relationship between purified versions of these helicases and their ability to regulate D‐loop stability in vitro [55, 132, 133]. A key in this model is the differentiation between nascent and extended D‐loops, with Sgs1 and Mph1 impacting nascent D‐loops [132], and Srs2 and Mph1 impacting extended D‐loops [134, 135].

Importantly, the composition of the proteins within the D‐loop likely impacts the rate of disruption. For example, both Mph1 and Sgs1 can disrupt D‐loops that are occupied by Rad51 and Rad54 [132]. In contrast, Srs2 is inhibited in the presence of Rad54 and Rdh54 [55], consistent with an earlier or later role of Srs2. Therefore, it is likely that the size of the recombinase filament and the extent of base pairing between donor and recipient DNA will impact the rate of general reversal.

While Sgs1 facilitates the reversal of D‐loops, it also prevents ectopic repair during both mitotic and meiotic recombination, suggesting its ability to reverse D‐loops is a regulatory role [136, 137]. Sgs1's role in D‐loop reversal is likely related to its ability to prevent ectopic or inappropriate recombination. However, the physical mechanism behind D‐loop reversal remains unclear. There is evidence that both the helicase activity of Sgs1 and the topoisomerase activity of Top3 are required for reversal [137, 138]. In bacteria, RecQ helicase limits the length of D‐loop structures, suggesting that it may regulate nucleotide pairing tracts [139]. A simple way to regulate the outcome would be to push DNA branches formed during strand invasion toward the 3’ end of DNA, as has previously been suggested [22, 24]. However, the general structure of a D‐loop intermediate allows for additional contributions from Top3‐Rmi1.

Top3 is a type 1A topoisomerase that can relax negative turns in supercoiled DNA [140]. In the absence of a decatenation loop, the enzyme is more likely to relax negatively supercoiled DNA. However, in the presence of a decatenation loop, the enzyme can unhook two concatenated pieces of DNA [28]. Eukaryotic Rmi1 contains a decatenation loop that can line the Top3 gate and stabilize its open conformation so that Top3 can use its topoisomerase activity to decatenate linked DNA molecules during dissolution of recombination intermediates in classical DSB repair [27, 138]. This activity participates in the dissolution of joint molecules but limits the ability of the topoisomerase activity to relax negatively supercoiled DNA. It is currently unclear whether the decatenation activity or the relaxation activity of Top3‐Rmi1 is required to reverse ectopic repair sites or D‐loops [33, 131]. Resolving this question will require the separation of these two activities.

Interestingly, the STR complex does not work on recombinase filaments composed of Dmc1. It has recently been shown that Mer3 competes with Sgs1 for binding to the Top3‐Rmi1, and this association with Mer3 may help recruit the D‐loop reversal activity of Top3‐Rmi1 to Dmc1‐mediated D‐loop intermediates [141]. Regulation of these intermediates is likely to change in the context of mismatched heteroduplexes between the donor and recipient DNA. However, this remains unknown.

### 1.7 Do Not Copy Your Neighbor

**FIGURE 3:**
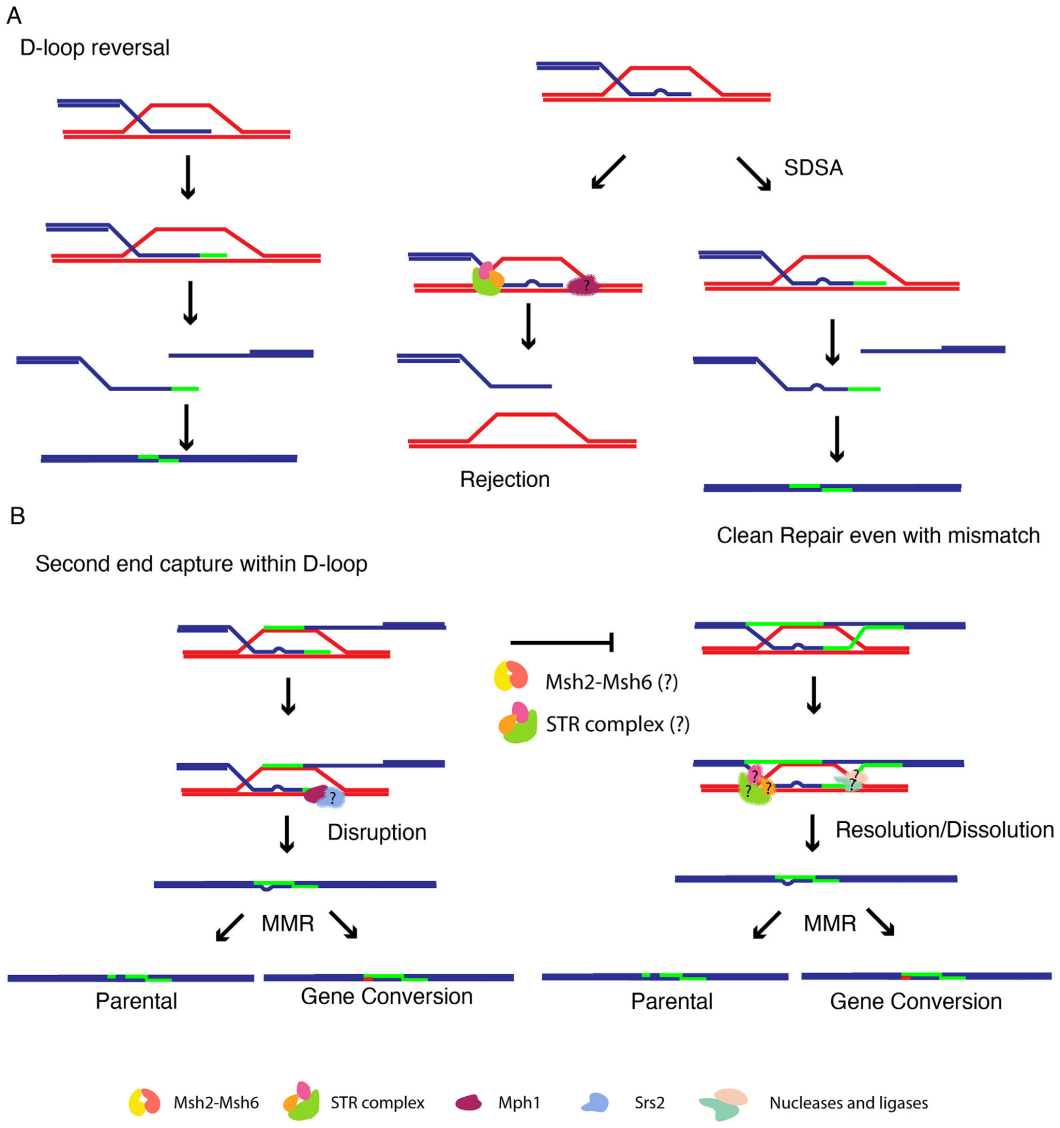
Possible strand pairing mismatch scenarios. (A). Comparisons for D‐loop reversal in the promotion of SDSA in the presence or absence of mismatched pairing nucleotides. This is an example in which the second end of DNA is captured outside of the D‐loop (B). Comparison of potential outcomes for mismatches that occur in the primary D‐loop when the extended D‐loop captures the second DNA end. Proteins shown with question marks have mechanistic question about their involvement during that HR step.

To understand the impact of mismatches and the need for D‐loop reversal in the context of a three‐strand intermediate, we need to evaluate the likelihood of a mismatch resulting in genomic changes during repair. Put a different way, the evolutionary need for a system will depend on the challenge it presents to growth. For example, repair outcomes that occur through SDSA will not incorporate the mismatched nucleotide into the repair product if mismatches only occur during strand pairing. This happens because the primary D‐loop is disrupted before the second end of the DNA is captured. Therefore, replication to complete repair will use the original recipient DNA to finish synthesis, resulting in seamless repair (Figure 3A) [8]. In contrast, a mismatch incorporated during DNA synthesis will persist after second end capture. As the synthesis proceeds, post‐replicative repair in favor of the recipient strand is silent, while repair in favor of the original donor strand can result in gene conversion. However, this is not impacted by Rad51/RecA‐mediated strand exchange and will not be recognized as a mismatch in a primary strand exchange intermediate.

Pairing‐dependent issues can arise if the primary D‐loop intermediate is not disrupted before second end capture. If the primary D‐loop has a mismatch, then the outgoing strand will also be homoeologous to the recipient DNA sequence. Therefore, replicating from the outgoing strand will cause a mismatch when the product is resolved or dissolved (Figure 3B). The mismatch in the repaired strands will then undergo repair through classical mismatch repair. Therefore, the resulting repair has a chance of inserting the donor nucleotide into the repair product, leading to a gene conversion event.

It is currently unclear whether there is a mechanism that prevents the second end of DNA from replicating the mismatch, or whether there is always a chance of a mismatch in this context. Candidates for proteins that may prevent incorporation of the mismatch include the Msh2‐Msh6 complex. Previous work has shown that deletion of *MSH6* in budding yeast results in increased HR outcomes, including increased gene conversion tract length in non‐crossover events and increased crossover events [142]. Msh2‐Msh3, Pms1, and Mlh2 deficiencies in yeast have also been shown to increase the length of gene conversion tracts relative to WT [143, 144, 145]. These results have been inferred to mean that MMR proteins may act as a physical barrier, with MSH proteins having a more drastic effect than MLH proteins [146]. The barrier might be formed by an increased Msh2‐Msh6 dwell time on the mismatch. This is enhanced through an interaction with the MLH proteins, blocking the extension of the 3’ end if the process is conserved well from prokaryotes [121, 122, 147].

It is also unclear if there are direct contributions of recombinase enzymes to the maturation of D‐loop structures, and how that will impact second end capture within the D‐loop or after disruption. The STR complex may restrict the size of D‐loops by destabilizing Rad51 through its helicase activity or topoisomerase activity. Sgs1 has been shown to promote BIR‐based repair [17, 18] when the second end of DNA has limited homology. Functionally, this could result from disruption in the primary D‐loop that will promote an SDSA‐like mechanism when the second end is long enough. This could occur through the removal of excess Rad51 [148], direct disruption of paired product, or both [149]. It is possible that Msh2‐Msh6 could increase the motor activity of Sgs1 to promote faster disruption when a mismatch is detected. This could restrict second end capture to outside of the parent D‐loop, promoting SDSA. This idea remains speculative, and future work should be focused on testing this relationship.

Extensive work on heteroduplex rejection in the context of a primary three‐strand intermediate has been made using a BIR‐based recombination system. The pairing mismatches that occur during BIR may be problematic if strand recognition occurs in an ectopic region of the genome. In this case, replication can incorporate the wrong information into a broken chromosome, resulting in insertions or rearrangements [150]. This problem can be avoided if replication tracts are kept short, which may be related to recombinase stability at the primary D‐loop. Pairing mismatches on primary strand exchange using a BIR system have provided direct evidence that the thermodynamics of base pairing dictate stable pairing and repair synthesis [151]. The context of a DNA mismatch during the primary exchange intermediate is likely to affect when and where the mismatch repair machinery enters the HR pathway. The role of the MMR machinery activity can be divided into how DNA mismatches are dealt with, between pre‐ and post‐elongation stages of HR [121, 142]. Studies using a BIR system have shown that if a mismatch is recognized before the start of synthesis, the ratio of mismatches to the tract length of homology and the existence of a nonhomologous tail are key to the decision to reject the heteroduplex [152]. Therefore, limiting the amount of contact between the donor and recipient DNA improves the overall fidelity of the recombination reaction. In all these cases, high fidelity is maintained when the pairing between donor and recipient DNA is kept short, preventing the need to disassemble or reverse complex strand exchange intermediates. Strand exchange events that are initiated by Rad51 tend to promote these short pairing tracts.

### 1.8 To all Good Things, an End

In general, homologous recombination can be considered a high‐fidelity DNA repair pathway. For the most part, this is achieved because the pathway is meticulous in picking sites with high homology in the early steps of the pathway and reduces the prevalence of complex DNA intermediates. The fidelity of the overall mitotic and meiotic HR reactions may occur through distinct pathways and may be dependent on their respective evolutionary goals. Mitotic HR is a dedicated DNA repair pathway that benefits from limited contact between chromosomes. In contrast, meiotic HR is designed to align chromosomes, which likely benefit from the extensive primary contacts formed during pairing. Both pathways act to regulate the stability of chromosomes within organisms. Future studies should be focused on understanding how differences between these two systems evolved to achieve similar, yet unique goals.

## Author Contributions

**Ilayda Korkmaz** and **J. Brooks Crickard** Co‐wrote the Manuscript.

## Funding

This work is supported by NIGMS R35142457 and American Cancer Research Scholar Grant RSG‐25‐1410641‐01‐DMC to JBC.

## Conflicts of Interest

The authors declare no conflicts of interest.

## Data Availability

Data sharing not applicable—no new data generated.
